# Retraction Note: Social media usage and students’ social anxiety, loneliness and well-being: does digital mindfulness-based intervention effectively work?

**DOI:** 10.1186/s40359-025-02810-0

**Published:** 2025-05-07

**Authors:** Li Sun

**Affiliations:** School of Marxism, Zhoukou Vocational and Technical College, Zhoukou, 466000 China

**Retraction Note: BMC Psychology (2023) 11:362**

10.1186/s40359-023-01398-7

The editor and the publisher have retracted this article. The article was submitted to be part of a guest-edited issue. An investigation by the publisher found a number of articles, including this one, with a number of concerns, including but not limited to compromised editorial handling and peer review process, inappropriate or irrelevant references or not being in scope of the journal or guest-edited issue. Based on the investigation’s findings the editor therefore no longer has confidence in the results and conclusions of this article.

The author has not responded to correspondence regarding this retraction.

